# Postoperative cognitive change after cardiac surgery predicts long‐term cognitive outcome

**DOI:** 10.1002/brb3.1750

**Published:** 2020-07-17

**Authors:** Kristiina Relander, Marja Hietanen, Kirsi Rantanen, Juhani Rämö, Antti Vento, Kari‐Pekka Saastamoinen, Risto O. Roine, Lauri Soinne

**Affiliations:** ^1^ Clinical Neurosciences, Neuropsychology University of Helsinki and Helsinki University Hospital Helsinki Finland; ^2^ Clinical Neurosciences, Neurology University of Helsinki and Helsinki University Hospital Helsinki Finland; ^3^ Department of Cardiac Surgery, Heart and Lung Center University of Helsinki and Helsinki University Hospital Helsinki Finland; ^4^ University of Turku and Turku University Hospital Turku Finland

**Keywords:** cognition disorders, cognitive dysfunction, coronary artery bypass, neuropsychological tests, risk factors

## Abstract

**Objectives:**

Postoperative cognitive dysfunction (POCD) is a common consequence of coronary artery bypass grafting. However, domain‐specific associations between postoperative changes and long‐term performance are poorly known. The aim of this study was to investigate whether domain‐specific cognitive changes after cardiac surgery predict long‐term cognitive outcome.

**Materials and Methods:**

We assessed 100 patients (86 men, mean age 60) before coronary artery bypass grafting, with re‐examinations after one week, three months, and a mean of 6.7 years. The extensive neuropsychological test battery was organized into seven functional cognitive domains. Cognitive decline and improvement were defined with the reliable change index derived from 17 matching healthy controls. Analyses were adjusted for baseline cognitive performance, age, gender, education and cardiovascular risks factors.

**Results:**

On group level, one week after surgery 71% patients showed cognitive decline and 9% improvement in any functional domain, as compared to preoperative results. Three months postsurgery, decline was observed in 47% and improvement in 25% of patients. Executive functioning was the most sensitive domain to both decline and improvement. Postoperative dysfunction predicted long‐term cognitive deterioration six years after operation, particularly in the domain of executive functioning.

**Conclusions:**

POCD after coronary artery bypass grafting is an essential risk factor for long‐term deterioration and an indication for neuropsychological follow‐up. Assessment of change in executive functioning after coronary artery bypass grafting may help to identify patients at risk for unfavorable long‐term outcome.

## Graphical abstract

The aim of this study was to investigate whether domain‐specific cognitive changes after cardiac surgery predict long‐term cognitive outcome. We assessed 100 patients before coronary artery bypass grafting, with re‐examinations after one week, three months, and six years. Postoperative dysfunction predicted long‐term cognitive deterioration six years after operation, particularly in the domain of executive functioning.

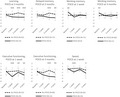

## 1 INTRODUCTION

Cognitive decline after coronary artery bypass grafting (CABG) has received a great deal of attention during the last few decades. Many surgery‐related factors, such as neuroinflammation, embolic load, cerebral blood flow, temperature management, glucose homeostasis, neurotoxicity of anesthetics and the use of cardiopulmonary bypass, have been suggested to contribute to postoperative cognitive dysfunction (POCD) after cardiac surgery (Berger et al., 2018; Bhamidipati et al., 2017). However, also patient‐related factors, such as preexisting neurovascular disease, age, preoperative hypoperfusion, depressive symptoms, and cognitive function, seem to predict decline (Berger et al., 2018; Bhamidipati et al., 2017; Ho et al., 2004; Kozora et al., 2010; Messerotti Benvenuti, Zanatta, Longo, Mazzarolo, & Palomba, 2012; Newman et al., 2001; Patron, Messerotti Benvenuti, Zanatta, Polesel, & Palomba, 2013). Thus, it is evident that the adverse cognitive effects of CABG need to be viewed in light of the individual brain's susceptibility (Hogan, Shipolini, Brown, Hurley, & Cormack, 2013).

Postoperative cognitive deterioration seems mostly transient on a group level. The reported incidence of POCD is roughly 50% shortly after surgery, with a decreasing trend during the following months (Knipp et al., 2008; Newman et al., 2001; Toeg, Nathan, Rubens, Wozny, & Boodhwani, 2013), but methodological differences, such as choice of outcome measures and variable definitions of decline, affect its recognition and reported incidence (Bhamidipati et al., 2017). Some studies use a combination of single neuropsychological tests (Evered, Silbert, Scott, Maruff, & Ames, 2016; Knipp et al., 2008, 2017; Kok, Koerts, Tucha, Scheeren, & Absalom, 2017), posing a risk for false‐positive classifications especially with a higher number of tests (Lewis, Maruff, Silbert, Evered, & Scott, 2006a). Others rely on more conservative combined scores of either domain‐specific tests (Newman et al., 2001; Phillips‐Bute et al., 2006; Toeg et al., 2013) or an entire test battery (Evered et al., 2016; Kok et al., 2017), the latter potentially masking underlying domain‐specific changes and complicating interpretation of the results. Often the definition of POCD remains arbitrary with a cutoff relative to patients’ baseline, such as 1 or 2 standard deviations (*SD*) in at least two or three tests or a composite score (Knipp et al., 2008, 2017; Kok et al., 2017; Newman et al., 2001; Toeg et al., 2013). More recently, reliable change index (RCI) methods that account for normal variation and practice effects derived from a control group (Lewis, Maruff, & Silbert, 2004; Lewis, Maruff, Silbert, Evered, & Scott, 2006b; Rasmussen et al., 2001) have gained popularity (Bruce, Yelland, Smith, & Robinson, 2013; Evered et al., 2016; Inouye et al., 2016).

Long‐term persistence of POCD, suggested by earlier uncontrolled studies (Newman et al., 2001), seems less probable than transient decline, as corroborated by more recent studies. According to a meta‐analysis, the initial decline observed less than two weeks after CABG surgery does not generally prevent regaining baseline level of performance by three months after surgery (Cormack et al., 2012). With respect to long‐term effects, cognitive performance in a cross‐sectional study 7.5 years after operation did not differ between patients that were randomized to either off‐pump CABG or angioplasty (Sauër et al., 2013). Neither were cognitive differences found between surgically and nonsurgically treated coronary artery disease patients after six years’ follow‐up (Selnes et al., 2009), which would suggest long‐term cognitive deterioration from underlying vascular or neurodegenerative disease rather than surgery. However, as Berger et al. (2018) point out, similar long‐term trajectories may also be related to nonrandomized design, group‐averaging that potentially masks decline in a subset of patients, counterbalanced positive and negative effects of coronary surgery, or a selective dropout of the most cognitively impaired (Berger et al., 2018).

Postoperative cognitive dysfunction should not be overlooked as a transient phenomenon. It has been shown to be related to decreased quality of life (Phillips‐Bute et al., 2006) although preoperative cognitive status may be a better predictor of life quality (Messerotti Benvenuti et al., 2013; Messerotti Benvenuti, Patron, Zanatta, Polesel, & Palomba, 2014). Postoperative cognitive dysfunction is also associated with increased mortality (Evered et al., 2016; Kok et al., 2017). Furthermore, the questions on long‐term deterioration and the association with specific cognitive domains are open. Some studies have found an association between short‐term POCD and future cognitive decline after an interval of several years. A decline of more than 1 *SD* compared with the patients’ baseline in any of four cognitive domains at discharge was related to a similarly defined cognitive decline and to change in a composite cognitive score five years later (Newman et al., 2001). Deterioration of more than 1 *SD* compared with the patients’ baseline in at least three individual tests at discharge was related to the incidence of cognitive decline three years after surgery (Knipp et al., 2008). In a more recent controlled study, a decline of reliable change index score exceeding 1.96 in at least two individual tests or in a composite score at three and 12 months was related to the occurrence of a similarly defined decline after a follow‐up time of 7.5 years (Evered et al., 2016). In conclusion, there is some evidence for associations between short‐ and long‐term declines after CABG, but the functional domains that have most prognostic long‐term value are yet to be studied.

Coronary surgery has also been proposed to have a favorable effect on cognition, possibly resulting from improved health or reduced need for medications (Berger et al., 2015). Although several studies and a meta‐analysis have shown improvement (Cormack et al., 2012), controlled studies suggest a generally equal groupwise improvement in surgical and nonsurgical patients, thus presumably reflecting learning in repeated testing (McKhann et al., 2005). However, some controlled studies have shown postoperative cognitive improvement (POCI) after CABG beyond learning effects. In a large epidemiological study, younger (<70 years) patients performed better than their twins or nonsurgical controls in a cross‐sectional telephone‐based cognitive screening test (Potter, Plassman, Helms, Steffens, & Welsh‐Bohmer, 2004). Surgical patients showed significantly greater improvement than their nonsurgical controls up to three years after surgery in verbal memory, but no other cognitive domains (Selnes et al., 2005). In a small controlled study using the reliable change index methodology, as many as 25% of bypass patients showed cognitive improvement in more than 20% of single cognitive tests eight weeks after operation, in contrast to 13% in a surgical control group (Bruce et al., 2013). Nevertheless, studies addressing cognitive improvement after CABG are scarce, and research on domainwise improvement is lacking.

To date, it is not known which cognitive functions have most prognostic value on long‐term cognitive outcome. This information would improve clinical cognitive assessment of POCD and identification of individuals susceptible to long‐term cognitive deterioration. The present study aimed to investigate the incidence of domain‐specific cognitive decline and improvement after CABG and their association with long‐term cognitive outcome.

## 2 MATERIALS AND METHODS

### 2.1 Patients

**FIGURE 1:**
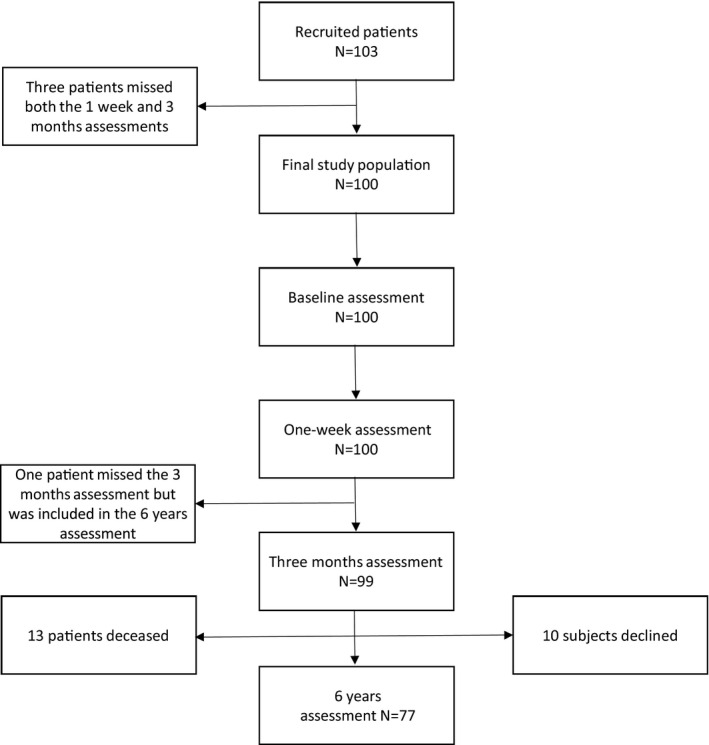
Assessed patients at each measurement

The study was approved by the local ethical committee and performed in accordance with the ethical standards of the Declaration of Helsinki. The subjects gave their informed consent prior to their inclusion in the study. We recruited 103 consecutive, independent, consenting patients who were to undergo a standard elective coronary artery bypass grafting with cardiopulmonary bypass (CPB) in the Department of Thoracic and Cardiovascular Surgery at the Helsinki University Hospital between 2.4.1995 and 29.3.1996. Patients with recent (within 6 months) cerebrovascular events or any other disability, which would be expected to interfere with the cognitive assessment, independence, or compliance (such as neurological disability or psychoses), were excluded. Three patients missed both the one‐week and three‐month assessments and were excluded. The final patient group consisted of 100 patients (86 men, mean age 60.4 years; Figure 1).

Educational qualifications were scored on a three‐code scale according to the Finnish education system: basic level (compulsory education requiring 6–9 years of education), middle level (vocational training, matriculation examination, and/or bachelor's degree requiring 8–15 years of education), or higher level (university‐level master's degree or higher requiring a minimum of 16 years of education). Occupational attainment was scored using a simple three‐code scale: physical or manual routine workers, qualified manual workers, and nonmanual white‐collar workers.

### 2.2 Control population

Control population consisted of 17 volunteers (12 men, mean age 63.6 years) with no medication, sign or history of cardiovascular, neurological, or neuropsychiatric morbidity, excessive alcohol intake over longer periods, or family history of neurodegenerative diseases. Age, gender, and educational or occupational level of the control population did not significantly differ from the patient population (Table 1).

**TABLE 1:** Baseline characteristics of the study population

Characteristic	Patients	Controls	*p*‐Value
Age, years	60.4 ± 8.6	63.6 ± 6.9	.15
Gender, male	86 (86%)	12 (71%)	.15
Education			
Basic level	44 (44%)	7 (41%)	.45
Middle level	48 (48%)	7 (41%)	
Higher level	8 (8%)	3 (18%)	
Occupation			
Manual routine	39 (39%)	4 (24%)	.45
Qualified manual	40 (40%)	8 (47%)	
Nonmanual	21 (21%)	5 (29%)	

Note

Data are presented as mean ± *SD* or *N* (%). *p*‐Values are from independent‐samples *t* test, chi‐square tests, or Fisher's exact test.

### 2.3 Surgical management

The patients were operated with standardized methodology. In CPB, a membrane oxygenator was used, the nonpulsatile pump flow was kept at 2.4 L/min/m^2^, the perfusion pressure was kept between 50 and 80 mmHg, and the core temperature was kept at 35°C. Ischemia time and cardiopulmonary bypass time were recorded. Serum concentration of neuron‐specific enolase (NSE) was determined from venous samples collected before surgery and controlled at 24 and 48 hr after surgery. NSE was analyzed with a time‐resolved fluoroimmunoassay (DELFIA^®^ NSE Kit, Wallac Oy, Turku, Finland). Change between baseline and 24 or 48 hr were used as variables.

### 2.4 Risk factor assessment

Smoking was measured in pack‐years (smoking years*amount of smoked packs of cigarettes per day). Alcohol consumption was assessed as alcohol units per week and categorized to less or more than 10 units per week. Mood was screened with the short 13‐item version of Beck Depression Inventory (BDI; Beck & Beck, 1972). DNA was extracted, and Apo‐E4 genotyping was done for 82 patients as described previously (Kontula, Aalto‐Setälä, Kuusi, Hämäläinen, & Syvänen, 1990). In addition, body mass index (BMI), preoperative glucose level, and occurrence of high blood pressure, diabetes, and dyslipidemia were recorded.

### 2.5 Neuropsychological assessment

The patients underwent a comprehensive neuropsychological assessment on the day before surgery, one week (range 6–8 days, mean 7 days), three months (range 80–112 days, mean 93 days), and six years (range 6.1–7.4 years, mean 6.7 years) after the operation. One patient missed the three‐month assessment. At the long‐term follow‐up, 13 patients had died, ten subjects declined, and 77 (77%) were examined (Figure 1). The control population was examined at approximately similar time intervals except for the late follow‐up.

Neuropsychological testing was performed by either of two certified neuropsychologists during a single visit. All repeated assessments were conducted by the same examiner. The test battery, shown in Table 2, was selected from established neuropsychological tests, following mostly the recommendations of consensus statement for cardiac surgery (Murkin, Newman, Stump, & Blumenthal, 1995). In attempt to minimize false‐positive rating due to multiplicity of tests (Bhamidipati et al., 2017) and to acquire domain‐specific information, a broader range of tests was used and classified into seven functional domains: learning, delayed memory, working memory, executive functioning, motor dexterity, processing speed, and reasoning (reasoning was not assessed at the three‐month assessment). In addition, general cognitive screening was done using Mini‐Mental State Examination (MMSE). The test battery was performed in the same order and approximately the same daytime for each participant. No participants had to discontinue testing because of fatigue. In order to minimize learning effects, parallel memory test versions were used in repeated measurements.

**TABLE 2:** Neuropsychological test battery

Cognitive domain	Test
Learning	Logical memory, Rivermead Behavioral Memory Test
	Auditory Verbal Learning Test (10 words, sum of trials 1–5)
	Rey Visual Learning Test (15 drawings, sum of trials 1–5)
Delayed memory	Delayed recall of logical memory
	Delayed recall of Auditory Verbal Learning Test
	Delayed recall of Rey Visual Learning Test
	Recognition of Rey Visual Learning Test
Working memory	Digit span forward
	Digit span backward
Executive functioning	Letter cancellation test (time to complete)
	Trail Making Test, part B subtracted by part A (times to complete)
	Stroop test, Word subtest subtracted by Color subtest (times to complete)
	Verbal phonemic fluency
	Verbal categorical fluency
Motor dexterity	Finger tapping, right hand
	Finger tapping, left hand
Processing speed	Trail Making Test, part A (time to complete)
	Stroop Color Subtest (time to complete)
Reasoning	Similarities, WAIS‐R
	Block Design, WAIS‐R
General cognitive screening	Mini‐mental state examination

All test scores were standardized using healthy controls' baseline performance as reference. Timed *z*‐scores were inverted so that negative *z*‐scores always indicated worse cognitive performance. Domainwise scores were formed by averaging the standardized *z*‐scores of each patient within each functional domain. Reflection and logarithmic transformation were applied to executive functioning and speed domain variables, reflection and square root transformation to reasoning and square root transformation to motor dexterity.

### 2.6 Statistical analysis

Statistical analyses were performed with IBM SPSS 25. *p*‐values below .05 were considered significant. Univariate groupwise comparisons were made between patients and controls (demographical characteristics), between late follow‐up patients and dropouts, and between patients that did or did not show POCD (demographical characteristics, baseline cognitive performance, surgery‐related factors, and cardiovascular risk factors). Independent‐samples *t* tests or Mann–Whitney *U* tests were used for continuous variables, and chi‐square tests or Fisher's exact tests for categorical variables. These p‐values were not corrected for multiple comparisons.

Preexisting cognitive impairment was defined as below 2 *SD* performance relative to the control group's baseline. Within‐domain performance in repeated testing was assessed with linear mixed models adjusting for age, gender, education, diabetes, high blood pressure, hyperlipidemia, smoking, and preoperative depressive symptoms. Because of collinearity, defined as variance inflation factor >5, occupation was not included in the analyses. Baseline, one‐week, and three‐month within‐domain *z*‐scores of patients and controls were used as dependent variables. Measurement served as within‐subject repeated effect, and based on Bayesian information criterion, compound symmetry was chosen for repeated effect covariance structure. Group (patients vs. healthy controls), measurement, and group*measurement were considered as potential predictors. Post hoc analyses of significant interactions were performed with pairwise comparisons between groups within each measurement and within groups between each measurement. The comparisons were based on estimated marginal means and were Bonferroni‐corrected.

The presence of POCD/POCI at one week and three months was determined using the reliable change index method in order to account for normal variation and practice effects (Lewis et al., 2004, 2006b; Rasmussen et al., 2001). The criterion of POCD/POCI was set to domain‐specific *z*‐score ± 2.

Associations between one week and three months postoperative cognitive dysfunction or improvement and within‐domain long‐term cognitive functioning were assessed with multiple linear regression analyses controlling for age, gender, education, domain‐specific baseline cognitive functioning, diabetes, high blood pressure, hyperlipidemia, smoking, and preoperative depressive symptoms. Due to high collinearity, defined as variance inflation factor >5, occupation was not included in the analyses. Then, other potential confounders (alcohol consumption, BMI, Apo‐E4 genotype, cardiopulmonary bypass time, ischemia time, rise in NSE level 24 and 48 hr after surgery, and preoperative glucose level) were entered into the models individually. Confounders with *p *< .2 were subsequently entered into the models simultaneously and checked for collinearity. Lastly, nonsignificant confounders were removed from final multivariate models. Local effect sizes were measured with Cohen's *F* ^2^ based on the R squares of the models (Cohen, 1988; Grissom, Kim, & Kim, 2012). Linear mixed models with Bonferroni‐corrected post hoc pairwise comparisons between groups (POCD vs. no POCD) were used to determine in which measurements the groups with and without POCD differed from each other. The associations between postoperative change in MMSE score (minimum three‐point decline from baseline) and domain‐specific long‐term cognition were also assessed with multiple regression analyses controlling for age, gender, education, and domainwise baseline performance.

## 3 RESULTS

### 3.1 Patient and control characteristics

Characteristics of the patient and control groups are shown in Table 1, with no significant differences between the groups. The patients of the long‐term follow‐up were slightly younger than dropouts (mean age 59.4 vs. 63.9 years, *p* = .02 in independent‐samples *t* test), but there were no differences in gender, education, occupation, surgery‐related factors, cardiovascular risk factors, domainwise POCD occurrence, or baseline MMSE score between late follow‐up patients and dropouts. However, the dropouts performed more poorly at baseline in learning (*p *= .02, *U* test) and executive functioning (*p *= .04, *U* test). These data are presented in detail in Table S1.

### 3.2 Differences in cognitive functioning between patient and control groups

Out of the 100 patients examined at baseline, the number of patients that performed below −2 *SD* compared with controls was two patients in learning, four in delayed memory, two in working memory, 26 in executive functioning, four in speed, two in motor dexterity, and 18 in reasoning. None of the controls performed below −2 *SD* in any of the cognitive domains.

**FIGURE 2:**
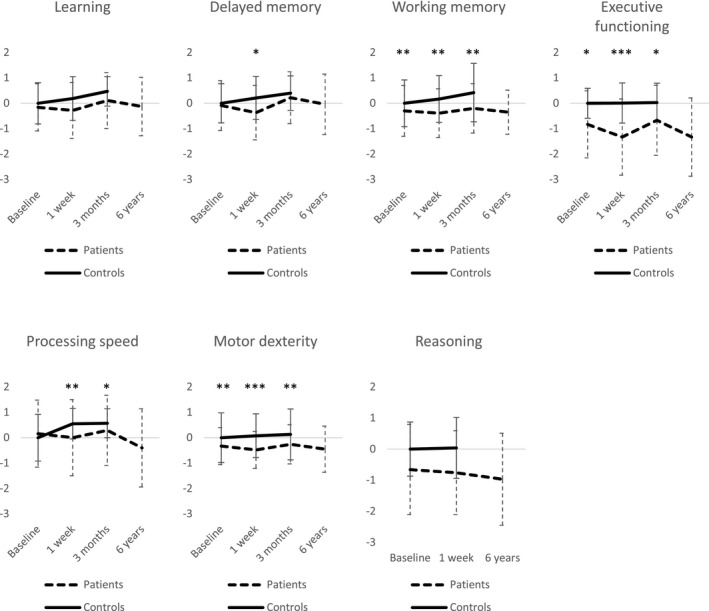
Time‐course of groupwise cognitive performance in each cognitive domain expressed as mean *z*‐scores. Error bars reflect standard deviations. *p < .05, **p < .01, and ***p < .001 in linear mixed models (main effects or post hoc analyses of significant interactions) adjusted for age, gender, education, diabetes, high blood pressure, hyperlipidemia, smoking, and preoperative depressive symptoms

Cognitive performance of patients and controls is presented in Figure 2, Table 3, and Table S2. On group level, significant postoperative cognitive changes in patients compared with controls were shown mainly at one week after surgery followed by recovery at three months. Linear mixed models showed a significant group*measurement interaction in delayed memory (*F*(2, 228.03) = 5.14, *p*=.007), executive functioning (*F*(2, 229.16) = 3.05, *p *= .049), speed (*F*(2, 228.78) = 7.41, *p *< .001), and motor dexterity (*F*(2, 228.95) = 3.64, *p *= .028). The group*measurement interaction was not significant in learning, working memory, or reasoning, but there was a significant main effect of group in working memory (*F*(1, 106.01) = 8.38, *p *= .005).

**TABLE 3:** Cognitive performance of patients and controls in the preoperative, 1‐week, 3‐month, and 6‐year measurements

Cognitive domain	Patients				Controls		
	Baseline (*N* = 100)	1 week (*N* = 100)	3 months (*N* = 99)	6 years (*N* = 77)	Baseline (*N* = 17)	1 week (*N* = 17)	3 months (*N* = 17)
Learning	−0.16 (0.93)	−0.28 (1.10)	0.11 (1.10)	−0.13 (1.15)	0 (0.81)	0.19 (0.86)	0.47 (0.58)
Delayed memory	−0.09 (0.98)	−0.37 (1.07)	0.22 (1.02)	−0.04 (1.19)	0 (0.77)	0.21 (0.85)	0.40 (0.68)
Working memory	−0.30 (1.00)	−0.39 (0.96)	−0.20 (0.97)	−0.35 (0.87)	0 (0.92)	0.17 (0.92)	0.42 (1.15)
Executive functioning	−0.83 (1.32)	−1.33 (1.50)	−0.67 (1.38)	−1.33 (1.54)	0 (0.59)	0.01 (0.79)	0.03 (0.76)
Speed	0.16 (1.16)	0.04 (1.23)	0.29 (0.93)	−0.40 (1.45)	0 (0.92)	0.55 (0.60)	0.57 (0.57)
Motor dexterity	−0.33 (0.73)	−0.48 (0.73)	−0.26 (0.77)	−0.45 (0.91)	0 (0.98)	0.08 (0.86)	0.13 (1.00)
Reasoning	−0.66 (1.45)	−0.76 (1.35)		−0.97 (1.48)	0 (0.87)	0.04 (0.98)	

Note

Results are expressed as unadjusted, untransformed mean *z*‐scores (*SD*) standardized relative to the baseline of controls.

Between‐group post hoc analyses showed that there was a significant difference between patients and controls only at the one‐week measurement in delayed memory (*t*(133.70) = 0.63, *p *= .029). In speed, the difference between groups was more pronounced at one week (*t*(145.22) = 0.41, *p *= .004) than at three months (*t*(145.38) = 0.30, *p *= .032) and not significant at baseline. In executive functioning, the difference between patients and controls was stronger at one week (*t*(149.62) = 0.53, *p *< .001) than at baseline (*t*(149.62) = 0.33, *p *= .019) and three months (*t*(149.79) = 0.31, *p *= .029). Also in motor dexterity, patients and controls differed significantly at each measurement, but the difference was more pronounced at one week (*t*(124.39) = 0.25, *p *< .001) than at baseline (*t*(124.39) = 0.17, *p *= .007) and three months (*t*(124.47) = 0.19, *p *= .004).

Within patients, delayed memory differed significantly between baseline and one week (*t*(228.15) = 0.29, *p *< .001), between baseline and three months (*t*(228.15) = 0.30, *p *< .001), and between one week and three months (*t*(228.30) = 0.60, *p *< .001). In executive functioning, the one‐week assessment differed significantly from baseline (*t*(229.13) = 0.16, *p *< .001) and three‐month assessments (*t*(229.36) = 0.23, *p *< .001). In speed, there was a significant difference only between one week and three months (*t*(228.97) = 0.12, *p* = .003). In motor dexterity, the one‐week assessment differed significantly from baseline (*t*(228.94) = 0.05, *p *< .001) and three‐month assessments (*t*(229.04) = 0.07, *p *< .001). Within the control group, the assessments did not differ significantly in motor dexterity or executive functioning, but in delayed memory, there was a significant difference between baseline and three months (*t*(228.00) = 0.40, *p *= .030), and in speed, both the one‐week (*t*(228.76) = 0.29, *p *= .002) and three‐month (*t*(228.76) = 0.31, *p *= .002) assessments differed from baseline. Note that due to variable transformations, change in executive functioning, speed, and motor dexterity in post hoc tests is proportional instead of absolute.

### 3.3 Postoperative cognitive dysfunction

**FIGURE 3:**
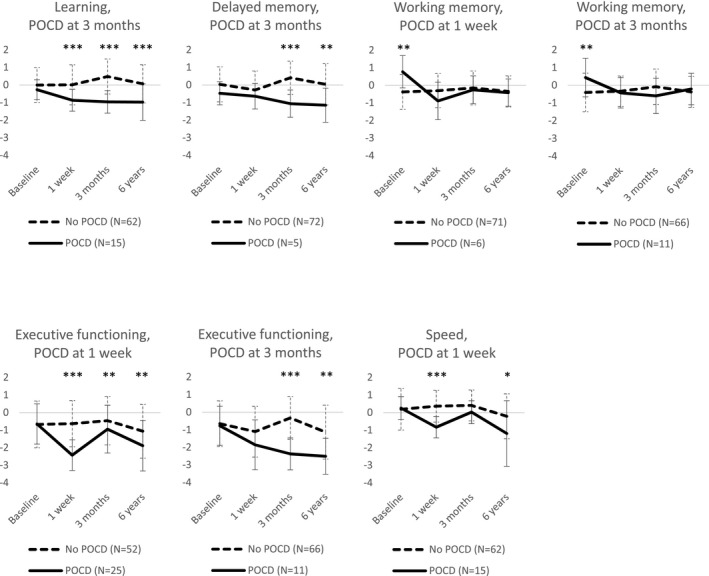
Cognitive performance of patients with and without domain‐specific postoperative cognitive dysfunction (POCD) in the domains and measurements that were associated with long‐term outcome. Results are expressed as mean *z*‐scores, and error bars reflect standard deviation. *p < .05, **p < .01, and ***p < .001 in post hoc analyses of linear mixed models

POCD in any of the cognitive domains at one week after surgery was evident in 55 (71%) of the 77 patients that also took part in six‐year assessment. Twenty‐five patients showed POCD in only one cognitive domain, 19 in two cognitive domains, and 11 in three to five cognitive domains. At three months, POCD in any of the cognitive domains was detected in 36 (47%) of the long‐term assessed patients. Twenty‐five patients showed POCD in only one cognitive domain, seven patients in two cognitive domains, and four patients in three to five cognitive domains. Executive functioning was most consistently affected by POCD (Table 4). One‐week POCD was significantly related to long‐term cognitive deterioration in working memory, executive functioning, and speed (small effect sizes), whereas POCD at three months was associated with long‐term decline in learning, delayed memory and working memory (small effect sizes), and executive functioning (medium effect size; Table 4). Time‐courses of cognitive performance in the domains and measurements that were associated with long‐term outcome in patients with or without POCD are shown in Figure 3. There were no significant associations between demographical characteristics, cardiovascular risk factors, or surgery‐related confounders and POCD in the most affected cognitive domain, executive functioning, at three months (Table 5).

**TABLE 4:** POCD occurrence and effects on long‐term cognitive performance

Cognitive domain	1‐week POCD			3‐month POCD		
	*N* (%)	Beta (*p*‐value)	Effect size	*N* (%)	Beta (*p*‐value)	Effect size
Learning	16 (21%)	NS		15 (20%)	−0.58 (.005)	0.13*
Delayed memory	17 (22%)	NS		5 (7%)	−0.82 (.011)	0.10*
Working memory	6 (8%)	−0.73 (.004)	0.14*	11 (14%)	−0.40 (.045)	0.06*
Executive functioning	25 (33%)	0.23 (.036)^a^	0.07*	11 (14%)	0.44 (<.001)^a^	0.20**
Speed	15 (20%)	0.26 (.016)^a^	0.09*	4 (5%)	NS	
Motor dexterity	10 (13%)	NS		8 (10%)	NS	
Reasoning	14 (18%)	NS				

Note:

Frequencies, unstandardized beta coefficients, *p*‐values, and effect sizes from linear regression analyses. Analyses are adjusted for age, gender, education, domain‐specific baseline cognitive functioning, diabetes, high blood pressure, hyperlipidemia, smoking, and preoperative depressive symptoms. Other potential confounders did not reach significance and are not included in final models. Effect sizes are *F* ^2^ values according to Cohen (1988): * small (>.02), ** medium (>.15), and *** large (>.35).

Abbreviations: NS, not significant; POCD, domain‐specific postoperative cognitive dysfunction.

^a^ Due to variable transformation, beta coefficients of executive functioning and speed are inversed in sign and indicate proportional instead of absolute decrease.

**TABLE 5:** Differences between patients with or without POCD at 3 months in executive functioning

	Executive functioning		
	POCD (*N* = 13)	No POCD (*N* = 86)	*p*‐Value
Age, years	59.6 ± 10.0	60.5 ± 8.4	.74
Gender, male	12 (92%)	73 (85%)	.69
Education			
Basic level	9 (69%)	34 (40%)	.11
Middle level	4 (31%)	44 (51%)	
Higher level	0 (0%)	8 (9%)	
Occupation			
Manual routine	8 (62%)	30 (35%)	.21
Qualified manual	3 (23%)	37 (43%)	
Nonmanual	2 (15%)	19 (22%)	
Smoking, pack‐years	3 (17)	15 (32)	.19
Alcohol, >10 units/week	0 (0%)	7 (8%)	.59
Body mass index	26.7 (3.8)	27.3 (5.0)	.75
Dyslipidemia	4 (31%)	48 (56%)	.09
High blood pressure	8 (62%)	47 (55%)	.64
Diabetes	2 (15%)	15 (17%)	1.0
Preoperative depression	4 (3)	4 (4)	.83
Baseline performance	−0.7 (1.7)	−0.5 (2.4)	.68
Apo‐E4 genotype			
1 allele	4 (31%)	16 (19%)	.14
2 alleles	1 (8%)	2 (2%)	
Cardiopulmonary bypass time	90 (28)	88.5 (36)	.86
Ischemia time	60.4 ± 16.9	61.6 ± 21.3	.85
Rise in 24 hr NSE level	7.2 (4.7)	7.4 (5.6)	.77
Rise in 48 hr NSE level	2.9 (1.9)	1.9 (4.2)	.31
Glucose level	5.8 (1.3)	5.9 (1.4)	.84

Note

Data are presented as mean ± *SD*, median (interquartile range), or *N* (%). *p*‐Values are from independent‐samples *t* tests, Mann–Whitney *U* tests, chi‐square tests, or Fisher's exact tests.

Abbreviations: NSE, neuron‐specific enolase; POCD, domain‐specific postoperative cognitive dysfunction.

On screening level, 15 patients showed a minimum three‐point drop in MMSE score at one week postsurgery and only four patients at three months postsurgery. MMSE drop in either measurement had no significant associations with long‐term performance in any cognitive domain.

### 3.4 Postoperative cognitive improvement

POCI at one week after surgery was shown in only seven (9%) of the 77 patients taking part in the six‐year assessment. Improved cognitive domains were working memory and executive functions (two patients in both domains), learning, speed, and motor dexterity (one patient in each domain). At three months, 19 (25%) patients showed POCI. It was most commonly shown in executive functions (8 patients), motor dexterity (6 patients), and delayed memory (4 patients), none of which had significant predictive value on long‐term cognitive functioning. POCI at three months was observed only in a single patient in learning, speed, and working memory.

## 4 DISCUSSION

The present study shows that while mostly transient at group level, the occurrence of POCD after CABG predicts cognitive deterioration six years after surgery. In contrast, POCI is rare and does not have the same long‐term prognostic value. The present findings suggest that POCD may be an important marker for the risk of long‐term cognitive deterioration, emphasizing the importance of recognizing it.

The cognitive domains with evident deterioration in the present study were delayed memory, executive functioning, speed, and motor dexterity. However, the groupwise decline was mainly detected shortly after surgery, and no longer observable three months postsurgery. Our results are consistent with earlier findings (Cormack et al., 2012) that POCD after CABG is, on group level, essentially transient and mostly not detectable by three months.

Our findings, nevertheless, stress the importance of recognizing the subset of patients who decline cognitively after CABG. POCD in any cognitive domain was manifest in 71% of patients one week postsurgery and in 47% three months postsurgery. Most notably, it was a significant predictor of cognition 6–7 years after surgery, even when confounders such as age, gender, education, baseline performance, cardiovascular risks factors and surgery‐related factors were taken into account. Given the relatively young age of the patients in the present study (considering the usual debut age of neurodegenerative diseases), it is plausible that the observed cognitive deterioration may proceed to clinically relevant decline and possibly dementia. POCD was associated with later cognitive decline but not dementia in an earlier study with a follow‐up of 7.5 years (Evered et al., 2016), and obviously, studies with longer follow‐up times and preferably larger samples are warranted.

Our study provides novel information on the cognitive domains that have prognostic long‐term value, in addition to confirming earlier notions of an association between POCD and long‐term deterioration (Evered et al., 2016; Knipp et al., 2008; Newman et al., 2001). The most consistently deteriorated cognitive domain one week and three months after surgery was executive functioning. Short‐term deterioration at one week was related to long‐term decline in working memory, executive functioning, and speed. Decline at a more stable phase at three months after surgery was related to long‐term deterioration in learning, delayed memory, working memory, and executive functioning. However, only a very small number of patients showed early decline in working memory or later decline in delayed memory. Moreover, the effect sizes of decline were generally small. Moderate effect size was found only in executive functioning and only at three months after surgery, indicating a greater predictive value when decline is observed at a later stage of recovery. Indeed, according to recent guidelines, POCD should only be specified later than 30 days after surgery, because earlier assessments may be affected by confounders such as emotional stress, pain, and medications (Evered et al., 2018). Our results also show that simple cognitive screening, such as MMSE, is not adequate for assessment of postoperative decline. Instead, executive functions seem most vulnerable to POCD and long‐term decline, probably because of the complex and widespread neurocognitive network they require. We suggest updating the recommendations on neuropsychological assessment methods after cardiac surgery (Murkin et al., 1995) in order to capture change in the cognitive domains that are most susceptible to change and have important predictive clinical value. Taken together, our results indicate that postoperative dysfunction in executive functioning three months after surgery is the most notable predictor of long‐term cognitive decline.

Postoperative improvement was found in a substantially lower proportion of patients (9% at one week and 25% at three months) than decline. The overall incidence of POCI at three months was comparable to that shown in a previous study (Bruce et al., 2013). Executive functioning was the most often improved cognitive domain. However, unlike POCD, the observed improvement was not associated with long‐term cognitive level. In accordance, earlier studies have shown cognitive improvement at three years (Selnes et al., 2005) but no longer six years after CABG (Selnes et al., 2009). It has been suggested that POCI is related to improved physical health and quality of life and that cognitive benefits diminish at the same pace that physical advantages of CABG are lost (Berger et al., 2015). Our results are in line with the view that POCI is transient and surpassed by other determinants of long‐term cognitive changes.

Strengths of this study include a long follow‐up time, the comprehensive neuropsychological test battery that allowed comparisons within several functional cognitive domains, and precise definition of postoperative dysfunction and improvement, including controlling for practice effects. However, in addition to the restrictions of sample size, there are limitations that should be taken into consideration. Firstly, 23% of patients dropped out of the long‐term assessment. The dropout rate was similar to previous long‐term coronary disease studies (Sauër et al., 2013; Selnes et al., 2009), and it is partly inevitable in longer follow‐up of patients with severe disease due to mortality. Of the survivors, 11% declined to participate in the long‐term follow‐up visit when contacted by phone, many because of geographical distance, mobility problems, or other inconvenience. Although dropouts and participants did not differ in POCD rate, surgery‐related factors, or cardiovascular risk factors, dropouts were slightly older than participants and their baseline performance was worse in the domains of learning and executive functioning. Nonetheless, even in consideration of the potential bias of selective attrition, we could demonstrate an association between POCD and long‐term cognitive outcome.

Secondly, the patients differed from healthy controls in baseline cognition, namely executive functioning, working memory, reasoning, and motor dexterity, although there were no differences in age, gender, education, or occupational level. Moreover, on individual level, the patients exhibited preexisting cognitive impairment frequently in executive functions and reasoning, possibly causing floor effect and underestimation of POCD in these domains. Differences in populations were expected because coronary artery disease is generally associated with an increased risk for lower cognition (Deckers et al., 2017; Eggermont et al., 2012). Baseline cognition has been shown to predict the effect of CABG on cognitive performance (Kozora et al., 2010). To allow for the possibility that baseline differences have an impact on the association between POCD and long‐term cognitive performance, we adjusted the analyses for domain‐specific baseline cognitive performance. Even with adjustment to the preoperative level, we could demonstrate that POCD has prognostic value on long‐term deterioration.

Thirdly, the healthy control group was not available at the late follow‐up. However, practice effects are not likely to bias the results of patients after an interval of several years. An alternative approach would have been either to use the reliable change index method with the short‐term performance of the controls as a reference also at long term (as, e.g., Evered et al., 2016), or to measure healthy controls also at long term. We chose to use the reliable change index method only at short term to avoid overestimating long‐term learning effects. The long follow‐up increases the risk of confounding elements, for example, cardiovascular health differences between patients and controls. Consequently, we focused on the within‐group repeated testing of the patients at the long‐term follow‐up.

In conclusion, the present study demonstrates that individuals with postoperative cognitive dysfunction after coronary artery bypass grafting are at a greater risk for poorer long‐term cognition although on group level the decline is largely transient. Thus, careful screening for emergent POCD and its subsequent follow‐up should not be overlooked. POCD should be considered an essential risk factor for long‐term decline and eventually an indication for neuropsychological follow‐up. Moreover, it is apparent that simple cognitive screening tests, such as the MMSE, are not sufficient to capture clinically relevant decline. At best, cognitive assessment with elements particularly probing executive functions should be applied pre‐ and postoperatively, preferably at a stable stage of recovery. In practice, widely used neuropsychological tests such as the Trail Making, Stroop, and Cancellation tests provide easily administrable means for assessment of postoperative cognitive dysfunction after coronary artery bypass grafting.

## CONFLICT OF INTEREST

The authors declare that they have no conflict of interest.

## AUTHOR CONTRIBUTION

Risto O. Roine, Lauri Soinne, and Marja Hietanen contributed to the conception and design of the study. Risto O. Roine, Juhani Rämö, Antti Vento, Lauri Soinne, Marja Hietanen, Kirsi Rantanen, and Kari‐Pekka Saastamoinen designed and put into practice the data acquisition. Kristiina Relander drafted the article and performed the data analysis. Marja Hietanen and Lauri Soinne critically revised the manuscript. All authors discussed the analyses, commented on the manuscript, and approved the final submitted version of the manuscript.

## Supporting information

Table S1‐S2

## Data Availability

Requests for sharing of data will be given individual consideration after additional approval for sharing by the local ethics committee.
