# Supplementary material for: Postoperative cognitive change after cardiac surgery predicts long‐term cognitive outcome
Source: Brain Behav. 2020 Jul 17;10(9):e01750. doi: 10.1002/brb3.1750 (PMC7507551; doi:10.1002/brb3.1750)
Supplement: Supplementary file 1 — Table S1‐S2 [file BRB3-10-e01750-s001.docx]

| **Supplementary table 1.** Differences between patients participating in the six-year measurement and dropouts. | | | |
| --- | --- | --- | --- |
|  | *Participating (N=77)* | *Dropouts (N=23)* | *p-value* |
| Age, years | 59.4 ± 8.5 | 63.9 ± 8.1 | 0.02* |
| Gender, male | 65 (84 %) | 21 (91 %) | 0.51 |
| Education |  |  | 0.74 |
| Basic level | 33 (43 %) | 11 (48 %) |  |
| Middle level | 37 (48 %) | 11 (48 %) |  |
| Higher level | 7 (9 %) | 1 (4 %) |  |
| Occupation |  |  | 0.53 |
| Manual routine | 28 (36 %) | 11 (48 %) |  |
| Qualified manual | 33 (43 %) | 7 (30 %) |  |
| Non-manual | 16 (21 %) | 5 (22 %) |  |
| Smoking, pack years | 12.0 (32) | 13.0 (24) | 0.71 |
| Alcohol, >10 units/week | 5 (7 %) | 2 (9 %) | 0.66 |
| Body mass index | 27.0 (4.9) | 27.5 (4.5) | 0.75 |
| Dyslipidemia | 42 (55 %) | 11 (48 %) | 0.57 |
| High blood pressure | 44 (57 %) | 11 (48 %) | 0.43 |
| Diabetes | 13 (17 %) | 4 (17 %) | 1.00 |
| Preoperative depression | 4 (4) | 4 (3) | 0.74 |
| Apo-E4 genotype |  |  | 1.00 |
| 1 allele | 18 (24 %) | 2 (29 %) |  |
| 2 alleles | 3 (4 %) | 0 (0 %) |  |
| Cardiopulmonary bypass time | 91.5 (28) | 84.0 (44) | 0.15 |
| Ischemia time | 61.1 (24) | 57.0 (30) | 0.18 |
| Rise in 24 h NSE level | 7.9 ± 5.8 | 7.8 ± 3.9 | 0.93 |
| Rise in 48 h NSE level | 2.0 (3.5) | 0.5 (5.2) | 0.51 |
| Glucose level | 6.0 (1.5) | 5.6 (1.0) | 0.76 |
| Preoperative performance |  |  |  |
| Learning | 0.1 (1.2) | -0.7 (1.5) | 0.02* |
| Delayed memory | 0.1 (1.4) | -0.2 (1.5) | 0.07 |
| Working memory | -0.3 (1.4) | -0.3 (1.4) | 0.71 |
| Executive functioning | -0.4 (2.0) | -1.6 (1.6) | 0.04* |
| Speed | 0.4 (1.0) | 0.2 (1.9) | 0.59 |
| Motor dexterity | -0.3 (1.0) | -0.4 (1.0) | 0.73 |
| Reasoning | -0.4 (1.6) | -0.4 (2.1) | 0.99 |
| MMSE | 28.0 (2) | 28.0 (3) | 0.15 |
| POCD at one week |  |  |  |
| Learning | 16 (21 %) | 4 (17 %) | 1.0 |
| Delayed memory | 17 (22 %) | 4 (17 %) | 0.77 |
| Working memory | 6 (8 %) | 2 (9 %) | 1.00 |
| Executive functioning | 25 (33 %) | 7 (30 %) | 0.85 |
| Speed | 15 (20 %) | 4 (17 %) | 1.00 |
| Motor dexterity | 10 (13 %) | 2 (9 %) | 0.73 |
| Reasoning | 14 (18 %) | 7 (30 %) | 0.25 |
| POCD at three months |  |  |  |
| Learning | 15 (20 %) | 5 (23 %) | 0.77 |
| Delayed memory | 5 (7 %) | 1 (5 %) | 1.00 |
| Working memory | 11 (14 %) | 2 (9 %) | 0.73 |
| Executive functioning | 11 (14 %) | 2 (9 %) | 0.73 |
| Speed | 4 (5 %) | 2 (9 %) | 0.61 |
| Motor dexterity | 8 (10 %) | 3 (14 %) | 0.70 |
| Data are presented as mean ± SD, median (interquartile range) or N (%). | | | |
| p-values are from independent samples t-tests, Mann-Whitney U tests, χ² tests or Fisher's exact tests. | | | |
| * p<.05. POCD, domain-specific postoperative cognitive dysfunction; NSE, neuron specific enolase. | | | |

| **Supplementary table 2.** Cognitive performance of patients and controls in individual neuropsychological tests. | | | | | | | | | | | | | | | |
| --- | --- | --- | --- | --- | --- | --- | --- | --- | --- | --- | --- | --- | --- | --- | --- |
|  | *Patients* | |  |  |  |  |  |  |  | *Controls* | |  |  |  |  |
| *Test* | *Baseline (N=100)* | | *1 week (N=100)* | | *3 months (N=99)* | | *6 years (N=77)* | |  | *Baseline (N=17)* | | *1 week (N=17)* | | *3 months (N=17)* | |
| Logical memory | 9.28 | (3.45) | 9.52 | (3.59) | 10.12 | (3.31) | 9.51 | (4.00) |  | 10.82 | (3.40) | 10.88 | (3.50) | 10.77 | (2.25) |
| AVLT | 39.33 | (5.11) | 37.74 | (6.77) | 39.52 | (6.15) | 39.03 | (6.08) |  | 38.76 | (4.67) | 37.94 | (4.88) | 43.12 | (4.34) |
| RVLT | 29.59 | (13.95) | 28.65 | (14.72) | 35.33 | (15.64) | 30.43 | (15.89) |  | 31.12 | (10.08) | 38.47 | (11.40) | 36.24 | (8.06) |
| Delayed recall of Logical memory | 6.88 | (3.68) | 7.95 | (3.79) | 8.52 | (3.52) | 7.95 | (4.40) |  | 9.29 | (3.62) | 8.71 | (3.64) | 9.82 | (2.48) |
| Delayed recall of AVLT | 6.39 | (2.73) | 5.53 | (2.69) | 6.60 | (2.40) | 6.95 | (3.20) |  | 6.06 | (2.56) | 5.41 | (2.60) | 7.29 | (2.64) |
| Delayed recall of RVLT | 7.98 | (3.53) | 7.09 | (3.77) | 9.10 | (3.60) | 7.30 | (3.85) |  | 7.59 | (2.50) | 9.59 | (3.26) | 8.82 | (2.46) |
| Recognition of RVLT | 25.49 | (3.26) | 23.93 | (3.84) | 26.11 | (3.39) | 25.51 | (3.96) |  | 25.47 | (2.40) | 26.53 | (2.43) | 26.59 | (2.24) |
| Digit span forwards | 5.56 | (1.31) | 5.57 | (1.04) | 5.63 | (1.05) | 5.44 | (0.99) |  | 5.65 | (1.06) | 5.65 | (1.12) | 6.18 | (1.47) |
| Digit span backwards | 4.33 | (1.22) | 4.13 | (1.26) | 4.48 | (1.30) | 4.35 | (1.11) |  | 4.88 | (1.05) | 5.24 | (1.15) | 5.24 | (1.15) |
| Letter Cancellation Test* | 232.70 | (66.28) | 266.78 | (81.85) | 233.44 | (64.21) | 264.21 | (92.45) |  | 231.59 | (50.73) | 234.24 | (48.96) | 216.76 | (37.86) |
| Trail Making test, B - A* | 124.74 | (89.34) | 156.97 | (97.67) | 117.15 | (88.86) | 140.75 | (87.37) |  | 63.76 | (21.17) | 75.59 | (42.80) | 72.00 | (44.70) |
| Stroop test, Word - Color* | 19.72 | (14.41) | 20.71 | (17.33) | 16.60 | (11.99) | 23.04 | (16.59) |  | 15.06 | (7.09) | 11.24 | (5.84) | 13.18 | (7.04) |
| Verbal phonemic fluency | 13.00 | (4.64) | 13.32 | (5.49) | 13.13 | (5.15) | 12.05 | (5.27) |  | 15.06 | (5.49) | 16.12 | (6.33) | 15.29 | (5.74) |
| Verbal categorical fluency | 19.96 | (6.01) | 18.53 | (6.24) | 19.78 | (6.33) | 17.84 | (6.63) |  | 21.18 | (6.81) | 20.76 | (6.46) | 20.76 | (6.46) |
| Finger tapping, right hand | 47.75 | (7.32) | 46.19 | (7.26) | 48.37 | (7.66) | 46.30 | (8.99) |  | 50.79 | (9.25) | 51.35 | (8.77) | 51.82 | (10.18) |
| Finger tapping, left hand | 42.99 | (6.75) | 41.81 | (7.30) | 43.61 | (7.12) | 42.27 | (9.07) |  | 45.91 | (9.03) | 46.85 | (7.70) | 47.18 | (8.83) |
| Trail Making test, A* | 45.15 | (21.00) | 45.91 | (20.67) | 41.98 | (17.81) | 54.53 | (27.24) |  | 44.12 | (11.60) | 34.94 | (10.26) | 35.53 | (10.46) |
| Stroop Color* | 16.80 | (6.00) | 18.35 | (8.28) | 16.90 | (4.78) | 19.49 | (7.97) |  | 20.47 | (9.02) | 17.71 | (4.44) | 16.88 | (3.18) |
| Similarities, WAIS-R | 10.99 | (3.30) | 11.04 | (2.86) |  |  | 10.71 | (2.83) |  | 13.00 | (1.54) | 12.24 | (1.95) |  |  |
| Block Design, WAIS-R | 26.76 | (8.87) | 25.00 | (9.19) |  |  | 23.79 | (10.97) |  | 26.82 | (7.51) | 31.12 | (7.75) |  |  |
| Mean raw test scores (SD). AVLT, Auditory verbal learning test; RVLT, Rey visual learning test. *Lower score represents better performance. | | | | | | | | | | | | | | | |
